# A Mouse Model of Enterovirus D68 Infection for Assessment of the Efficacy of Inactivated Vaccine

**DOI:** 10.3390/v10020058

**Published:** 2018-01-30

**Authors:** Chao Zhang, Xueyang Zhang, Wenlong Dai, Qingwei Liu, Pei Xiong, Shuxia Wang, Lanlan Geng, Sitang Gong, Zhong Huang

**Affiliations:** 1 Joint Center for Infection and Immunity, Guangzhou Institute of Pediatrics, Department of Gastroenterology, Guangzhou Women and Children’s Medical Center, Guangzhou Medical University, Guangzhou 510623, China; chaozhang@ips.ac.cn (C.Z.); genglan_2001@hotmail.com (L.G.); 2 Unit of Vaccinology & Antiviral Strategies, CAS Key Laboratory of Molecular Virology & Immunology, Institut Pasteur of Shanghai, Chinese Academy of Sciences, University of Chinese Academy of Sciences, Shanghai 200031, China; xyzhang@ips.ac.cn (X.Z); wldai@ips.ac.cn (W.D.); qwliu@ips.ac.cn (Q.L.); pxiong@ips.ac.cn (P.X.); sxwang@ips.ac.cn (S.W)

**Keywords:** enterovirus D68, acute flaccid myelitis, mouse model, inactivated vaccine, neutralizing antibody

## Abstract

In recent years, enterovirus D68 (EVD68) has been reported increasingly to be associated with severe respiratory tract infections and acute flaccid myelitis (AFM) in children all over the world. Yet, no effective vaccines or antiviral drugs are currently available for EVD68. Although several experimental animal models have been developed, immunogenicity and protective efficacy of inactivated EVD68 vaccines has not been fully evaluated. To promote the development of vaccines, we established an Institute of Cancer Research (ICR) suckling mouse model of EVD68 infection in this study. The results showed that ICR neonatal mice up to about nine days of age were susceptible to infection with EVD68 clinical strain US/MO/14-18947 by intraperitoneal injection. The infected mice exhibited progressive limb paralysis prior to death and the mortality of mice was age- and virus dose-dependent. Tissue viral load analysis showed that limb muscle and spinal cord were the major sites of viral replication. Moreover, histopathologic examination revealed the severe necrosis of the limb and juxtaspinal muscles, suggesting that US/MO/14-18947 has a strong tropism toward muscle tissues. Additionally, β-propiolactone-inactivated EVD68 vaccine showed high purity and quality and induced robust EVD68-specific neutralizing antibody responses in adult mice. Importantly, results from both antisera transfer and maternal immunization experiments clearly showed that inactivated EVD68 vaccine was able to protect against lethal viral infection in the mouse model. In short, these results demonstrate the successful establishment of the mouse model of EVD68 infection for evaluating candidate vaccines against EVD68 and also provide important information for the development of inactivated virus-based EVD68 vaccines.

## 1. Introduction

Human enterovirus D68 (EVD68) is an emerging pathogen for acute respiratory infections and it is also linked to acute flaccid myelitis (AFM) [1]. Since its first identification in the United States of America (USA) in 1962 [2], EVD68 was seldom reported around the world until the early 2000s [1,3]. During the past decade, however, EVD68 infections have notably increased in North America, Europe and Asia [1,3,4]. In 2014, the USA experienced a large outbreak of severe respiratory disease caused by EVD68, which led to 1153 laboratory-confirmed cases including 14 deaths by January 2015 (https://www.cdc.gov/non-polio-enterovirus/about/ev-d68.html#outbreak). Recent epidemiological studies have revealed that EVD68 continues circulating in various parts of the world after 2014 [5,6,7,8,9]. Thus, EVD68 has been increasingly recognized as a significant respiratory pathogen in children. Furthermore, in the second half of 2014, 120 pediatric patients with confirmed AFM, characterized by acute limb weakness with spinal cord motor neuron injury, were registered in the USA; this AFM cluster was coincident with the EVD68 outbreak, suggesting a possible causal association between EVD68 infection and AFM [10,11]. This speculation was further confirmed by a number of subsequent case reports and epidemiological investigations [12,13,14,15,16].

EVD68 belongs to the D species of the *Enterovirus* genus in the *Picornaviridae* family. It is a non-enveloped RNA virus with an icosahedral capsid composed of four structural proteins (VP1, VP2, VP3, and VP4) [17]. Phylogenetic analysis of EVD68 *VP1* sequences revealed the presence of three main clades (A, B and C), which are circulating globally [3]. Clade B can be further classified into three subclades (B1, B2, and B3) [18]. In addition, EVD68 2014 outbreak isolates associated with AFM belong to subclade B1 [19].

So far, there are no effective vaccines or antiviral drugs for EVD68. A suitable animal model of EVD68 infection is required to help develop antiviral agents and vaccines and/or to study the pathogenic mechanisms. Previously Patel et al. found that four to six week-old cotton rats were permissive to transient EVD68 replication following intranasal infection; however, the infectious virus was cleared from the nose and lung tissues 48 h post infection, and no clinical symptoms or death were observed in the rats [20]. Similarly, intranasal infection of EVD68 resulted in minimal clinical symptoms in ferrets [21]. Therefore, the two animal models are not suitable for evaluation of EVD68 vaccines and antiviral agents. Recently, Hixon et al. reported that neonatal Swiss Webster mice developed a paralytic disease resembling human AFM after intramuscular or intracerebral infection with EVD68 clinical isolates, and further demonstrated that the paralysis was associated with infection and loss of motor neurons in the spinal cord [22]. However, characteristics of EVD68 infection in neonatal mice, including effect of age on susceptibility to infection, median lethal dose and viral loads and pathological changes of various tissues, have not been adequately identified. Moreover, immunogenicity and protective efficacy of inactivated EVD68 vaccines has not been evaluated. In the present study, we established a model of EVD68 infection by intraperitoneal inoculation of Institute of Cancer Research (ICR) suckling mice with specific EVD68 clinical strain, systematically analyzed the pathological characteristics of EVD68 infection in mice. Furthermore, we prepared and characterized β-propiolactone-inactivated EVD68 vaccine and then evaluated its protective efficacy using the established mouse model.

## 2. Materials and Methods

### 2.1. Cells and Viruses

Human rhabdomyosarcoma cells (RD; ATCC #CCL-136) were grown as described previously [23]. EVD68 prototype strain Fermon (GenBank ID: AY426531), and two EVD68 2014 outbreak isolates US/MO/14-18947 (GenBank ID: KM851225) and US/KY/14-18953 (GenBank ID: KM851231) were obtained from ATCC and grown in RD cells. EV71 strain EV71/G082 and CVA16 strain CVA16/SZ05 were described previously [23,24]. All viruses were titrated for the 50% tissue culture infectious dose (TCID_50_) in RD cells, using the Reed–Muench method [25].

### 2.2. Antibodies

Polyclonal antibodies against VP0, VP1 or VP3 proteins of EVD68 strain Fermon were described previously [26]. An anti-EVD68 monoclonal antibody (mAb) 6A11 was generated in our laboratory from mice immunized with inactivated US/MO/14-18947 using previously described protocols [23].

### 2.3. Mouse Infection Experiments

All animal studies were approved by the Institutional Animal Care and Use Committee at the Institut Pasteur of Shanghai on 17 February 2016, and the project identification code was 170071A. Mice were obtained from Shanghai Laboratory Animal Center (SLAC, China).

To compare the virulence of different EVD68 strains, one-day-old ICR mice were injected intraperitoneally (i.p.) with 2.0 × 10^6^ TCID_50_ of Fermon, US/MO/14-18947 or US/KY/14-18953. All mice were monitored daily for survival and clinical score for 14 days. Clinical scores were graded as follows: 0, healthy; 1, lethargy and reduced mobility; 2, limb weakness; 3, limb paralysis; 4, death.

To assess the effect of age on susceptibility to infection, ICR mice were injected i.p. with the most lethal EVD68 strain (2.0 × 10^6^ TCID_50_/mouse) at 1, 5, 7, 9 or 12 days of age. The infected mice were observed daily for survival and clinical score for 14 days using the same criteria as above.

To determine the effect of virus dose on mortality, ICR mice at the most sensitive age were inoculated i.p. with the most virulent strain of EVD68 at different doses (1.0, 30, 7.8 × 10^3^, 1.2 × 10^5^, and 2.0 × 10^6^ TCID_50_/mouse). After infection, all mice were monitored daily for survival and clinical score for 14 days using the same criteria as mentioned above.

### 2.4. Determination of Viral Loads in Tissues of Infected Mice

One-day-old ICR mice were injected i.p. with culture medium or 1.2 × 10^5^ TCID_50_ of US/MO/14-18947. After infection, blood and organs/tissues (brain, heart, lung, intestine, liver, spleen, kidney, limb muscle, and spinal cord) were harvested from the mice daily until 7 days post-infection (dpi). Then total RNA was isolated from each sample using TRIzol reagent (Invitrogen, Carlsbad, CA, USA), and subsequently reverse transcribed using PrimeScript RT reagent Kit (Takara, Kusatsu, Shiga, Japan), to generate cDNA. For quantification, real-time PCR was carried out in the 7900HT Fast Real-Time PCR System (Applied Biosystems, Foster City, CA, USA) by using SYBR Premix Ex Taq kit (Takara, Japan) according to the manufacturer’s protocol. The primers are as follows: forward primer, 5′-CGAGAGCATCATCAAAACAGCGACC-3′; reverse primer, 5′-CACTGTGCGAGTTTGTATGGCTTCT-3′. *VP1* gene fragment of US/MO/14-18947 was cloned into the vector pET28b, yielding plasmid pET28b-VP1, which was used as a standard to determine the absolute copy numbers of EVD68.

### 2.5. Histopathological and Immunohistochemical Staining

One-day-old ICR mice were inoculated i.p. with culture medium or EVD68 strain US/MO/14-18947 (1.2 × 10^5^ TCID_50_/mouse). 7 days later, the mice were euthanized, and various organs/tissues (brain, liver, kidney, spleen, heart, lung, intestine, limb muscle, and juxtaspinal muscle) were collected from each mouse and fixed in 4% paraformaldehyde for 24 h. Then, the samples were dehydrated, embedded in paraffin and sliced into 4 μm sections. For histopathological examination, the sections were stained with hematoxylin and eosin (H&E) and coverslipped, followed by observation under a light microscope. For immunohistochemical analysis, the sections were dewaxed, rehydrated and microwaved in citrate buffer; then, the sections were blocked with 5% BSA in PBS for 1 h and incubated at 4 °C overnight with EVD68-specific mAb 6A11 (0.6 mg/mL, 1:200 dilution), followed by incubation with horseradish peroxidase (HRP)-conjugated anti-mouse IgG (Dako, Glostrup, Denmark) for 0.5 h; 3,3′-diaminobenzidine (DAB) substrate (Dako, Denmark) was added for color development; the sections were counterstained with hematoxylin, coverslipped, and examined under a light microscope.

### 2.6. Preparation of Inactivated EVD68

RD cells were grown to 80% confluency at 37 °C and then infected with EVD68 strain US/MO/14-18947 at 33 °C at a multiplicity of infection (MOI) of 0.01. EVD68 was collected from the culture supernatant at 3 dpi and inactivated with β-propiolactone (Wako, Saitama, Japan; 1:4000 dilution) for 24 h at 4 °C, followed by incubation at 37 °C for 1 h for hydrolysis of the β-propiolactone. Then EVD68 was precipitated with 10% polyethylene glycol 8000, and further purified by 20% sucrose cushion and 10–50% sucrose gradient ultracentrifugations according to protocols described in previous studies [27,28]. The gradient fractions that contained virus antigens were collected, concentrated by one more round of ultracentrifugation and resuspended in PBS. Total protein concentration of the purified inactivated EVD68 sample was determined by the Bradford assay. Western blotting of inactivated EVD68 was carried out as described previously [28] with polyclonal antibodies against EVD68 VP0, VP1 or VP3 proteins.

### 2.7. Mouse Immunization and Serum Antibody Measurement

To generate the experimental vaccines, purified inactivated US/MO/14-18947 (1 μg/dose) was mixed with the aluminum hydroxide adjuvant (Invivogen, San Diego, CA, USA; 500 μg/dose) by vortexing. PBS was formulated similarly with the adjuvant, and served as a control. 8 week-old female BALB/c mice (*n* = 6 in each group) were injected intraperitoneally (i.p.) with the experimental vaccines at weeks 0 and 3. Serum samples were obtained from each mouse at week 5, and the resultant sera were heat-inactivated (56 °C, 30 min) to destroy complement and used in subsequent experiments. Total anti-EV-D68 IgG antibodies in immune sera were measured using indirect ELISA as described previously [26]. Serum neutralizing antibody titers against EVD68 were detected by micro-neutralization assay as described previously [26]. The neutralizing titers were defined as the highest serum dilutions that completely inhibited appearance of cytopathic effect (CPE).

### 2.8. In Vivo Protection Assays

The protective efficacy of the inactivated EVD68 vaccine was tested using two different assays. In one assay, groups of naive ICR mice less than one day of age were i.p. injected with 20 µL of pooled anti-PBS or anti-US/MO/14-18947 sera. One day later, the suckling mice were inoculated i.p. with 1.2 × 10^5^ TCID_50_ of US/MO/14-18947. In another assay, groups of eight week-old female ICR mice were immunized twice with PBS (negative control) or inactivated US/MO/14-18947 as described above and allowed to breed after the last immunization. One-day-old pups born to immunized dams were infected i.p. with 1.2 × 10^5^ TCID_50_ of US/MO/14-18947. After challenge, all mice were monitored daily for survival and clinical score for 14 days using the same criteria as described above.

## 3. Results

### 3.1. Comparison of the Virulence of Different EVD68 Strains in Mice

Suckling mice are used in this study, because adult mice are not susceptible to EVD68 infection [20]. In addition, it is very difficult to perform intranasal infection in newborn mice, although the respiratory tract is the natural route of infection. Therefore, intraperitoneal (i.p.) injections, an easier and more common practice, were used instead.

**Figure 1:**
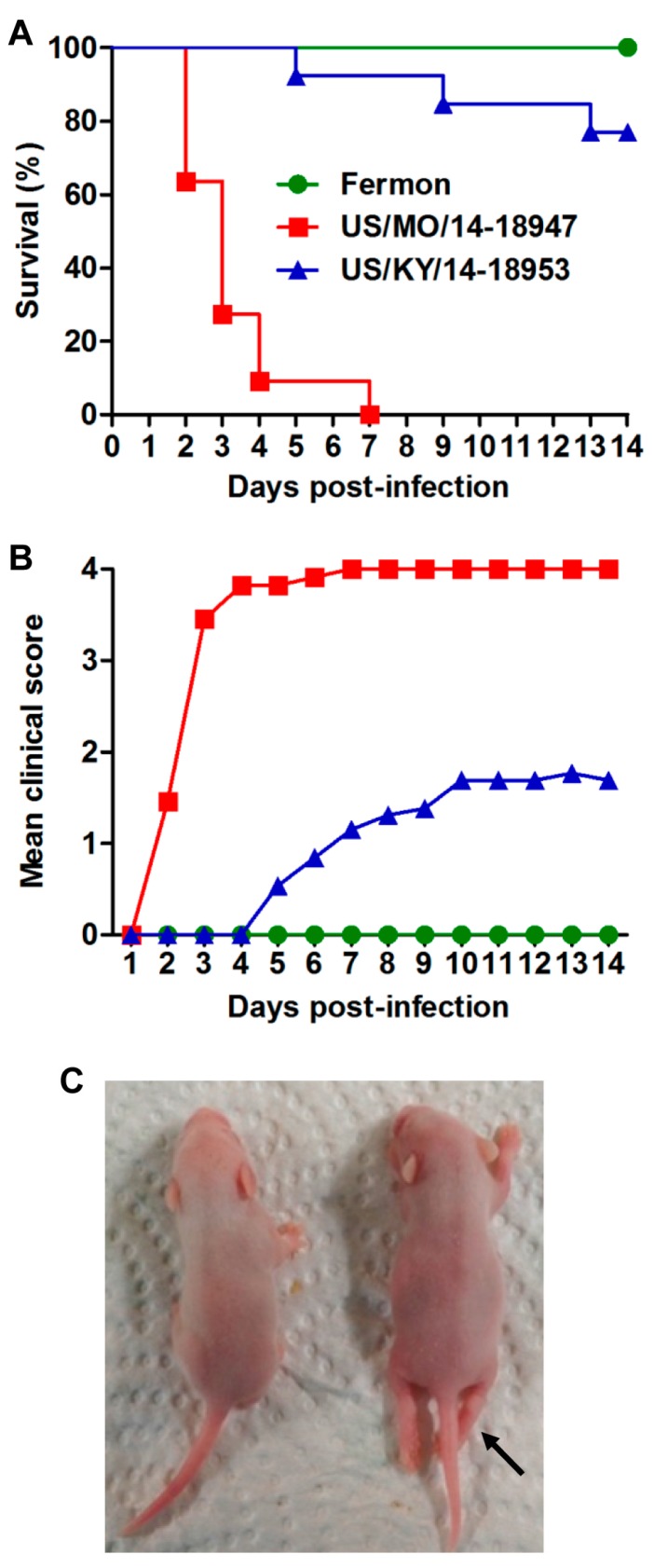
Comparison of the virulence of different EVD68 strains in mice. One-day-old ICR mice (*n* = 11–13/group) were inoculated i.p. with 2.0 × 10^6^ TCID_50_ of Fermon, US/MO/14-18947 or US/KY/14-18953. All mice were monitored daily for (**A**) survival and (**B**) clinical score for 14 days. Clinical scores were graded as follows: 0, healthy; 1, lethargy and reduced mobility; 2, limb weakness; 3, limb paralysis; 4, death; (**C**) a representative picture of hind limb paralysis caused by US/MO/14-18947 at 6 dpi is shown (arrow). The mouse on the left side is a naive age-matched control.

To compare the virulence of various strains of EVD68, groups of one-day-old ICR mice were inoculated i.p. with the same dose of the prototype strain Fermon, and 2014 USA outbreak isolates US/MO/14-18947 (clade B1) and US/KY/14-18953 (clade A), respectively. Clinical symptoms and survival were monitored daily after infection. As shown in Figure 1, mice infected with the Fermon strain showed no signs of disease and all survived; whereas US/MO/14-18947-infected mice exhibited limb paralysis and ultimately all died within 7 dpi. 62% of mice inoculated with US/KY/14-18953 developed clinical signs, including limb weakness and paralysis, and three of them eventually died with a final mortality rate of 23.1% (Figure 1A,B). These results indicate that US/MO/14-18947 is much more virulent for ICR mice than Fermon and US/KY/14-18953 strains. Therefore, EVD68 strain US/MO/14-18947 was selected as the challenge virus strain in this study.

### 3.2. US/MO/14-18947 Infection Induced Death in Mice in an Age- and Virus Dose-Dependent Manner

**Figure 2:**
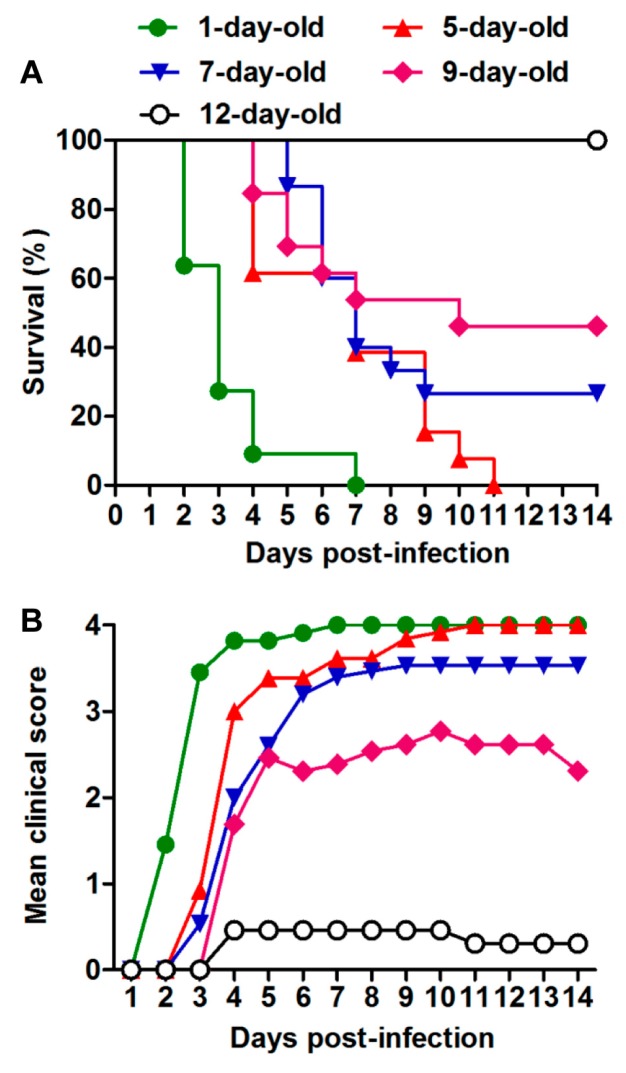
Age dependence of EVD68-induced disease and mortality. ICR mice (*n* = 11–15/group) were injected i.p. with 2.0 × 10^6^ TCID_50_ of US/MO/14-18947 per mouse at 1, 5, 7, 9 or 12 days of age. (**A**) Survival and (**B**) clinical score were then monitored and recorded daily after infection. Clinical scores were graded as described in the legend of Figure 1.

To determine the influence of age on the susceptibility of mice to EVD68 infection, ICR mice at different ages were injected i.p. with US/MO/14-18947 at 2.0 × 10^6^ TCID_50_ per mouse. As shown in Figure 2, one-day-old mice began to die at 2 dpi, and all were dead by 7 dpi. 5-day-old mice became sick at 3 dpi and eventually all died within 11 dpi. The mortality rates of mice in the 7- and 9-day-old groups dropped to 73% and 54%, respectively. All mice infected at 12 days of age survived, although three of them (23%) showed mild symptoms. These results indicate that the virulence of EVD68 strain US/MO/14-18947 for mice is age-dependent, with one-day-old mice being the most sensitive to infection. Therefore, one-day-old ICR mice were chosen as the animal model in this study.

**Figure 3:**
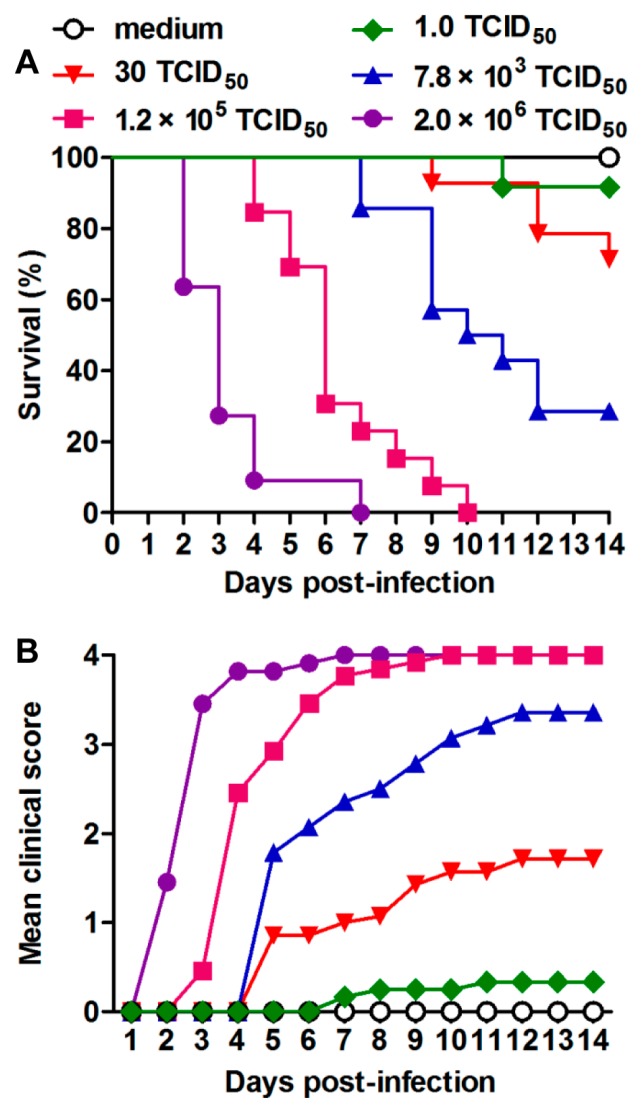
Dose dependence of EVD68-induced disease and mortality. One-day-old ICR mice (*n* = 11–14/group) were inoculated i.p. with increasing doses of US/MO/14-18947 (from 1.0 to 2.0 × 10^6^ TCID_50_/mouse). Control mice were given medium only. (**A**) Survival and (**B**) clinical score were monitored daily for 14 days following infection. Clinical scores were graded as described in Figure 1 legend.

To evaluate the effect of virus dose on severity of disease and mortality, various doses of US/MO/14-18947 were inoculated i.p. into one-day-old ICR mice. Figure 3 showed that all mice injected with doses of 2.0 × 10^6^ and 1.2 × 10^5^ TCID_50_/mouse were dead by 7 dpi and 10 dpi, respectively. With virus doses of 7.8 × 10^3^ and 30 TCID_50_/mouse, the mortality rates dropped to 71% and 29%, respectively. Only one out of 12 (8.3%) mice inoculated with a dose of 1 TCID_50_/mouse showed limb weakness at 7 dpi and eventually died. In contrast, the mice injected with medium alone (control group) did not show any signs of disease and all survived (Figure 3). These observations suggest that there is a significant positive correlation between the infectious dose of US/MO/14-18947 and the death rate of mice. Furthermore, the median lethal dose (LD_50_) was 2866 TCID_50_/mouse, which was calculated by GraphPad Prism software (version 5, GraphPad Software Inc, LA Jolla, CA, USA). A dose of 1.2 × 10^5^ TCID_50_/mouse (~42 LD_50_) was selected for subsequent viral challenge studies.

### 3.3. Tissue Viral Loads in US/MO/14-18947-Infected Mice

**Figure 4:**
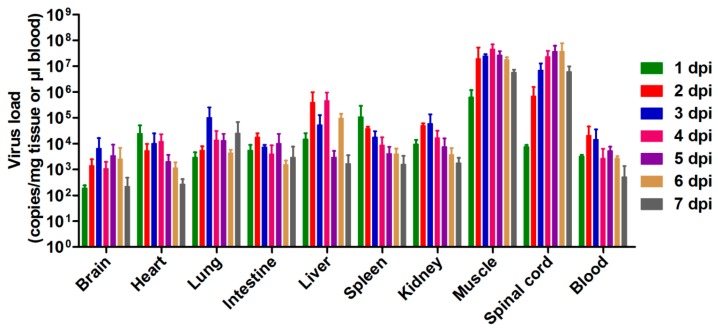
Viral loads within various tissues from EVD68-infected mice. One-day-old ICR mice were inoculated i.p. with 1.2 × 10^5^ TCID_50_ of US/MO/14-18947 per mouse. Tissues were collected every 24 h after infection and viral loads within the indicated tissues were determined by real-time quantitative RT-PCR. Results are expressed as viral RNA copies per milligram of tissue or per microliter of blood. Data represent the mean ± SD for three mice per group.

To survey the replication and distribution of EVD68 virus in infected mice, virus loads in various tissues from US/MO/14-18947-infected mice were determined by real-time PCR. Note that RNA titers are usually 10–100 times higher than infectious virus titers. As shown in Figure 4, 24 h after infection, the virus was detected in all organs and tissues tested, suggesting that the virus had already entered the bloodstream and spread around the body. The viral loads in the limb muscle and spinal cord increased gradually over time and reached the peaks at 4 and 6 dpi, respectively, and were significantly higher than those in other tissues (Figure 4). In contrast, viral loads were undetectable in all tissues of medium-inoculated mice. These findings indicated that the limb muscle and spinal cord were the major sites of viral replication.

### 3.4. Pathological Analysis of US/MO/14-18947-Infected Mice

**Figure 5:**
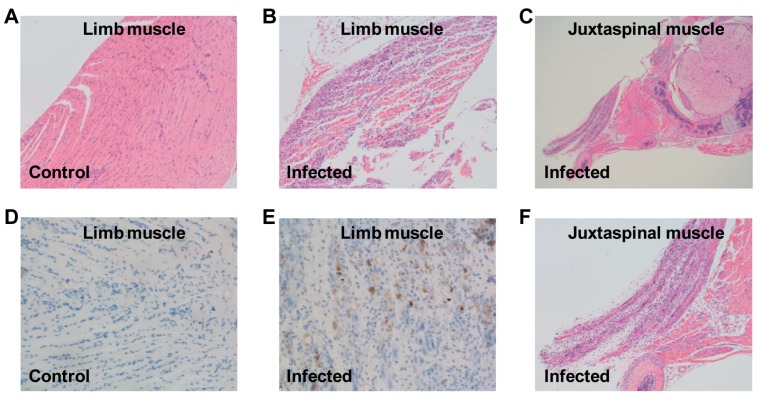
Histological examination of EVD68-infected mice. One-day-old ICR mice were inoculated i.p. with (**A**,**D**) culture medium or (**B**,**C**,**E**,**F**) 1.2 × 10^5^ TCID_50_ of US/MO/14-18947. Tissues were collected at day 7 after inoculation and then subjected to (**A**–**C**,**F**) H&E staining or (**D**,**E**) immunohistochemical staining with an anti-EVD68 mAb 6A11 as the primary antibody; (**F**) expanded view of panel (**C**). Magnification: panels (**A**,**B**,**F**): ×100; panel (**C**): ×40; panels (**D**,**E**): ×200. Images shown are representative of two ICR mice in each group.

To investigate the cause of limb paralysis and death, various tissues derived from US/MO/14-18947-infected mice (grade 3) or medium-injected mice (control) were subjected to histopathologic examination. H&E staining of infected mice revealed severe necrotizing myositis in the limb muscle compared to normal morphous from control mice (Figure 5A,B). Immunohistochemical results confirmed that viral antigens were present in the limb muscle of infected mice but absent in that of control mice (Figure 5D,E). In addition, severe necrosis was also found in the juxtaspinal muscle of infected mice (Figure 5C,F). These findings were in agreement with the results of viral load determination (Figure 4). However, no significant pathological changes were observed in other detected tissues of infected mice, including brain, liver, kidney, spleen, heart and intestine.

### 3.5. Evaluation of the Protective Efficacy of Inactivated EVD68 Vaccine in the Mouse Model

**Figure 6:**
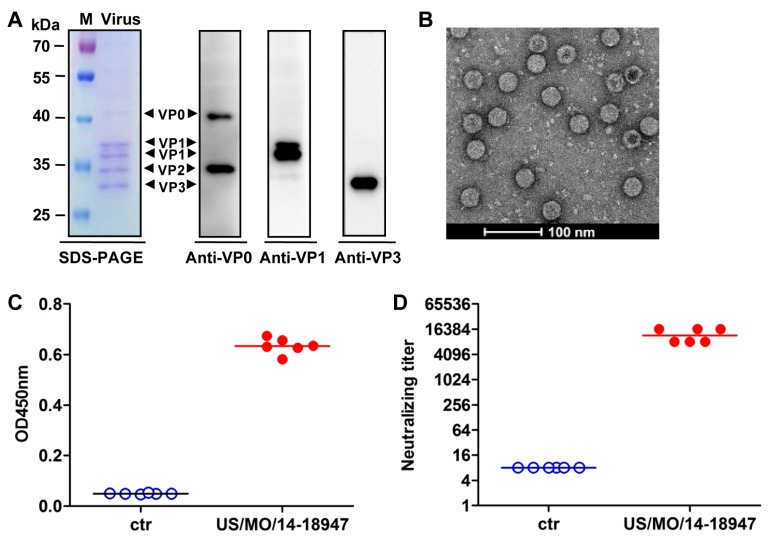
Neutralizing antibody responses induced by inactivated US/MO/14-18947 in adult mice. (**A**) SDS-PAGE and western blotting analysis of purified inactivated EVD68. Lane M, protein marker; lane Virus, inactivated US/MO/14-18947; (**B**) visualization of inactivated US/MO/14-18947 by electron microscopy; (**C**,**D**) serum antibody responses induced by inactivated US/MO/14-18947. Groups of six BALB/c mice were immunized twice with PBS (ctr) or inactivated US/MO/14-18947. Antisera were collected 2 weeks after the final immunization and used for different assays; (**C**) virus-specific antibody responses measured by ELISA with purified US/MO/14-18947 as the coating antigen. The antisera were diluted 1:1000; (**D**) neutralization titers against US/MO/14-18947. The antisera from PBS-immunized mice did not show any neutralization effect at the lowest dilution (1:16) and were assigned a titer of 8 for geometric mean titer (GMT) calculation. Each symbol represents a mouse, and the solid line indicates the geometric mean value of the group.

The application of this neonatal mouse model in vaccine evaluation was assessed using inactivated EVD68 vaccine. At first, the experimental EVD68 inactivated vaccine was prepared from US/MO/14-18947-infected RD cells. Results of SDS-PAGE and western blotting analyses explicitly revealed that EVD68 virus consisted of VP0, VP2, VP1 and VP3 proteins (Figure 6A). The subsequent electron microscopy analysis of the purified sample showed two kinds of 30-nm spherical particles, solid-core particles (full particles) and hollow-core particles (empty particles) (Figure 6B). To test the immunogenicity, two groups of BALB/c mice were injected twice with inactivated US/MO/14-18947 formulated with alum and PBS formulated with alum (negative control), respectively. The serum samples taken two weeks after the last immunization were used to detect virus-specific antibodies by ELISA with purified EVD68 viral particles as the coating antigen. As shown in Figure 6C, only background levels of binding were observed in the PBS group; in contrast, all sera from mice injected with inactivated US/MO/14-18947 exhibited significant reactivity. In addition, the micro-neutralization assay showed that all antisera from inactivated virus-immunized mice potently neutralized the homologous strain US/MO/14-18947 with a geometric mean titer (GMT) of 11,585; whereas the anti-PBS sera did not show any neutralization effect even at the lowest dilution (1:16) (Figure 6D). Moreover, the cross-neutralization test revealed that the pooled anti-US/MO/14-18947 sera could neutralize the heterologous strains Fermon and US/KY/14-18953, but did not display any neutralizing activity against other enteroviruses, including EV71/G082 and CA16/SZ05, at the minimum dilution ratio of 1:32 (Table 1). These results demonstrate that inactivated US/MO/14-18947 could elicit robust EVD68-specific neutralizing antibody responses in mice.

**Figure 7:**
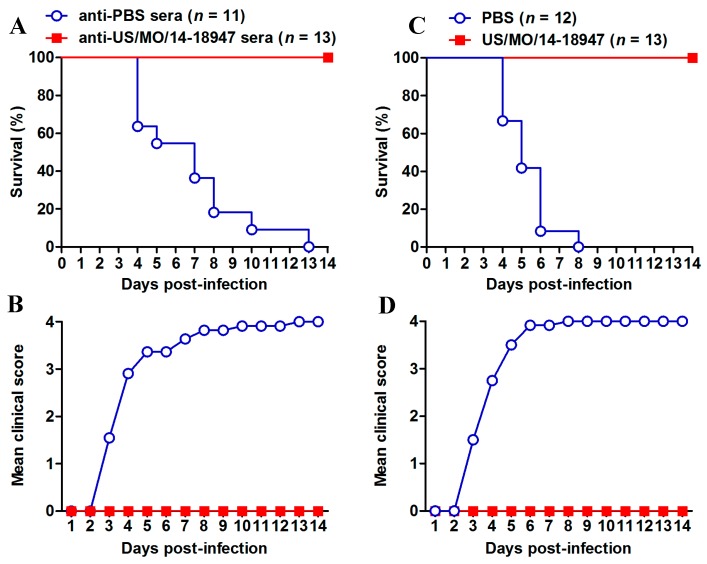
Protective efficacy of inactivated US/MO/14-18947 in the EVD68 infection mouse model. (**A**,**B**) Passive transfer of anti-US/MO/14-18947 sera conferred complete protection against lethal viral challenge in suckling mice. Groups of ICR mice less than one day of age were i.p. injected with anti-US/MO/14-18947 or anti-PBS sera. One day later, the mice were challenged i.p. with US/MO/14-18947 and then monitored daily for (**A**) survival and (**B**) clinical score for 14 days; (**C**,**D**) maternal immunization with inactivated US/MO/14-18947 fully protected suckling mice against lethal viral challenge. One-day-old ICR mice born to dams immunized with PBS or inactivated US/MO/14-18947 were infected with US/MO/14-18947, followed by daily observation of (**C**) survival and (**D**) clinical score for 14 days. Clinical scores were graded as described in the legend of Figure 1. The number of mice in each group is indicated in brackets.

The in vivo protective efficacy of the inactivated US/MO/14-18947 vaccine was evaluated in the mouse model established in this study. Groups of newborn ICR mice were i.p. injected with anti-US/MO/14-18947 or anti-PBS sera, followed by challenge with a lethal dose of US/MO/14-18947. As shown in Figure 7A,B, the mice receiving the anti-PBS sera began to show disease symptoms at 3 dpi and all of them eventually died within 13 dpi. In contrast, all of the anti-US/MO/14-18947 sera-treated mice survived and did not show any clinical signs during the two-week observation period. These results indicated that anti-US/MO/14-18947 sera could confer effective protection against lethal viral challenge in the mouse model.

We further tested whether the maternal immunity could provide protection against EVD68 infection in suckling mice. Neonatal ICR mice born to dams immunized with PBS or inactivated US/MO/14-18947 were infected with a lethal dose of US/MO/14-18947. As shown in Figure 7C,D, the mice in the PBS group became sick at 3 dpi and ultimately all died within 8 dpi. In contrast, all pups born to the inactivated US/MO/14-18947-immunized dams showed a 100% survival rate without overt clinical symptoms. Taken together, these data indicate that the animal model established here is applicable to the preclinical assessment of EVD68 vaccine candidates.

## 4. Discussion

EVD68 has recently emerged as an important cause of severe respiratory illness and neurologic disease, mainly AFM [1,29]. Therefore, it is of great significance to generate mouse models of EVD68 infection, which is helpful to evaluate vaccines and antiviral drugs against EVD68 and/or better understand pathogenesis. Recently, it was reported that EVD68 clinical isolates could cause limb paralysis in two-day-old Swiss Webster mice [22]. In this study, we also found that ICR neonatal mice up to about nine days of age were susceptible to EVD68 infection (Figure 2), suggesting that age, not mouse strain, is a limiting factor for efficient EVD68 infection in mice. We further demonstrated that EVD68 infection induced death in suckling mice in an age- and virus dose-dependent manner (Figure 2 and Figure 3). In addition, We found that i.p. injection of US/MO/14-18947 could result in 100% mortality of ICR mice (Figure 2 and Figure 3), contrary to Hixon et al.'s observation that i.p. injection of the same virus strain produced disease in only 4.5% of Swiss Webster mice [22], indicating that the i.p. route is sensitive for ICR mice, rather than Swiss Webster mice. Although i.p. injection is not the natural route of infection, the illness caused by infection resembled paralysis in humans (Figure 1C).

EVD68 infections primarily cause mild to severe respiratory illness; moreover, they have also been associated with AFM [1,29], which affects the nervous system, particularly the spinal cord [30]. The current study showed that the viral loads in the spinal cords of EVD68-infected mice increased significantly with time (Figure 4); whereas the viral loads in the brains of infected mice only slightly increased at 2 and 3 dpi, and were still considerably lower than those in the spinal cords (Figure 4), indicating that the spinal cord, not the brain, is the major site of viral replication, consistent with the previous finding that EVD68 could infect and kill motor neurons in spinal cord of neonatal mice [22]. Moreover, very high viral loads and obvious pathological changes were observed in the muscles of infected mice (Figure 4 and Figure 5), indicating that muscle tissue is another major location for viral replication. Altogether, these findings may partly explain the phenomenon of paralysis. In addition, the viral loads in the lungs of infected mice increased to some extent only at 3 dpi, but were still relatively low compared to those in the spinal cords and muscle tissues (Figure 4), suggesting that EVD68 virus has a weak tropism to the lung in the neonatal mouse and our mouse model of EVD68 infection is a model of paralytic myelitis rather than a pneumonia model.

The present study revealed that EVD68 clinical strains from the 2014 USA outbreak displayed significantly higher virulence than the prototype strain Fermon in neonatal mice (Figure 1). The increased viral virulence may be linked to the great differences in the amino acid sequences between EVD68 clinical strains and prototype strain. Further studies are needed to identify the key virulence determinants of EVD68.

In the present study, an experimental EVD68 inactivated vaccine was found to be highly immunogenic and capable of inducing high-titer neutralizing antibodies against homologous and heterologous EVD68 strains in adult mice (Figure 6 and Table 1). By employing the mouse model established in this study, we tested the in vivo efficacy of inactivated EVD68 vaccine. Our results demonstrated that both serum neutralizing antibodies and maternal antibodies, which were elicited by inactivated US/MO/14-18947 vaccine, were capable of conferring complete protection against lethal EVD68 infection in the suckling mouse model (Figure 7).

Generally, cell culture-derived enteroviruses exist in two forms of particles, full particles (with viral RNA genome) composed of VP4, VP2, VP1 and VP3 proteins and empty particles (without viral genome) composed of VP0, VP1 and VP3 proteins [31,32]. Furthermore, many previous studies have demonstrated that full particles can induce more potent neutralizing antibody responses than empty particles [31,32]. In this study, by electron microscopy analysis, we also observed the presence of two types of EVD68 particles (Figure 6B). Moreover, the high ratio of full particles to empty particles was consistent with our SDS-PAGE results that the content of VP2 protein was far more than that of VP0 protein (Figure 6A,B), and may also provide a partial explanation for the good immunogenicity of the EVD68 inactivated vaccines in mice.

In conclusion, we successfully established an ICR suckling mouse model of EVD68 infection for assessment of the protective efficacy of candidate vaccines against EVD68, and these results also provide valuable information for the development of inactivated whole virus-based EVD68 vaccines.

## Figures and Tables

**Table 1:** Cross-neutralization activity of the pooled antisera against heterologous strains and viruses.

Pooled Antisera	Neutralization Titer Against				
	EVD68/ Fermon	EVD68/ US/MO/14-18947	EVD68/ US/KY/14-18953	EV71/ G082	CVA16/ SZ05
Anti-PBS (control)	<32	<32	<32	<32	<32
Anti-US/MO/14-18947	128	8192	256	<32	<32

The lowest serum dilution tested is 1:32.
